# Larger cells have relatively smaller nuclei across the Tree of Life

**DOI:** 10.1002/evl3.243

**Published:** 2021-06-29

**Authors:** Martino E. Malerba, Dustin J. Marshall

**Affiliations:** ^1^ Centre of Geometric Biology, School of Biological Sciences Monash University Melbourne Australia; ^2^ Centre for Integrative Ecology, School of Life and Environmental Sciences Deakin University Victoria Australia

**Keywords:** Artificial selection, C‐value enigma, genome size evolution, karyoplasmic ratio, limiting pool hypothesis, nucleoskeletal theory, nucleotypic theory, optimal DNA theory, selfish DNA hypothesis

## Abstract

Larger cells have larger nuclei, but the precise relationship between cell size and nucleus size remains unclear, and the evolutionary forces that shape this relationship are debated. We compiled data for almost 900 species – from yeast to mammals – at three scales of biological organisation: among‐species, within‐species, and among‐lineages of a species that was artificially selected for cell size. At all scales, we showed that the ratio of nucleus size to cell size (the ‘N: C’ ratio) decreased systematically in larger cells. Size evolution appears more constrained in nuclei than cells: cell size spans across six orders of magnitude, whereas nucleus size varies by only three. The next important challenge is to determine the drivers of this apparently ubiquitous relationship in N:C ratios across such a diverse array of organisms.

**Impact Summary**

One of the oldest tenets of biology is that the ratio between the nucleus size and the cell size (the “N:C” or “karyoplasmic” ratio) is roughly constant. In this work, we challenged the tenet of a constant N:C ratio by comprehensively mapping these two fundamental traits across the Tree of Life. First, we compiled a massive among‐species dataset on cell size and nucleus size covering 879 species, ranging from microbes to mammals. Second, we assembled 7929 observations of both traits within‐species, ranging from yeast to plants and metazoans. Third, we artificially selected 72 lineages of a model unicellular eukaryote for smaller and larger cell sizes across 500 generations (ca. 3 years) and tracked the fate of the N:C ratios in thousands of cells. Our meta‐analyses revealed a previously unrecognized systematic pattern in N:C ratios at all biological scales: *Larger cells have relatively smaller nuclei across all scales of biological organization – from among‐species, to within‐species, to among‐lineages of a species that was artificially size‐selected*. We would argue that our discovery of a cryptic relationship between the two of the most fundamental units in biology – the cell and its nucleus – is of the broadest possible appeal. The patterns we present are unanticipated by theory and have implications for biomedicine, where the N:C ratio of a cell is a diagnostic tool for disease – including metastatic tumors. Future studies should investigate the evolutionary forces for such a predictable decrease in relative nucleus size with increasing absolute cell size and understand what this pattern means for cell functions.

Small cells have small nuclei, large cells have large nuclei. Cell biologists originally believed that the ratio of nucleus size to cell size (the “N:C” or “karyoplasmic” ratio) was essentially constant (Cavalier‐Smith 2005; Greilhuber *et al*. 2013; Vukovic *et al*. 2016), that is, every increase in cell size was matched By a proportional increase in nucleus size. Since then, it has become clear that although N:C ratios typically remain tightly controlled within a narrow range, they can still vary substantially (Jorgensen *et al*. 2007; Neumann and Nurse 2007; Hara and Merten 2015). While this variation in N:C ratios makes clear that cell size and nucleus size are not inexorably bound, our understanding of this fundamental anatomical relationship is hindered by the lack of a quantitative meta‐analysis.

Meanwhile, the various hypotheses that seek to explain the positive relationship between cell size and nucleus size make no predictions on how N:C ratios should vary. For example, the “limiting pool hypothesis” posits that the size of the nucleus is defined by the amount of local resources in the surrounding cell cytoplasm (Neumann and Nurse 2007). This theory implies a positive relationship between cell size and nucleus size, but makes no prediction regarding changes in N:C ratio nor on whether the relationship should be linear or nonlinear. Other hypotheses (e.g., nucleoskeletal theory, nucleotypic theory; (Gregory 2001; Cavalier‐Smith 2005) make similar conclusions – cell size and nucleus size should be positively related but say no more beyond this.

We argue that an essential first step is to determine how N:C ratios vary across the Tree of Life and, crucially, whether N:C ratios show any systematic patterns. Such patterns would provide essential clues as to the underlying drivers of the relationship between cell size and nucleus size. Unfortunately, formal evidence for trends in N:C ratios, both among‐ and within‐species, is actually remarkably scarce. For comparisons among species, there have been no formal statistical tests of this relationship, particularly tests that account for shared evolutionary lineages and phylogenetic non‐independence (Pagel and Johnstone 1992; Vinogradov 1999). For comparisons within species, evidence is scattered and piecemeal, with formal analysis of systematic trends lacking for most. Finally, very few studies have explored how microevolutionary shifts in the size of one component (e.g., the cell) influences the evolution of the other component (e.g., the nucleus). Estimating how these two components co‐evolve would elucidate the selective forces that shape the relationship between cell size and nucleus size.

We estimate N:C ratios at three scales of biological organization: among‐species, within‐species, and among lineages artificially selected for smaller and larger cell sizes. We compiled an among‐species dataset on cell size and nucleus size covering 879 species, ranging from prokaryotes to mammals and also including the nucleoid (c.f. nucleus) of prokaryotes. For the within‐species dataset, we compiled 7929 observations across 20 species, ranging from yeast to plants to metazoans. Finally, we evolved 72 lineages of the green alga *Dunaliella tertiolecta* for 500 generations (ca. 3 years), artificially selecting for different cell sizes while tracking the fate of their N:C ratios. Our results show that N:C ratios are not simply variable, but instead decline predictably with increasing cell size at all scales of biological organisation: larger cells almost invariably have relatively smaller nuclei.

## Results

### N:C RATIO AMONG SPECIES

**Figure 1:**
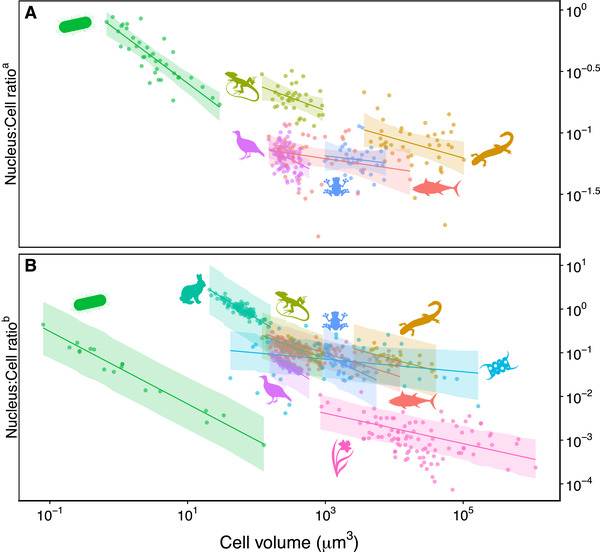
Among‐species comparisons of nucleus volume to cell volume ratio as a function of cell volume (all axes are log_10_‐transformed). Each dot represents a species, color‐coded for taxonomic clade: nucleoids of prokaryotes, and nuclei of phytoplankton, angiosperms, birds, amphibians (divided between frogs and salamanders), reptiles, fish, and mammals. Continues lines represent the clade‐specific model predictions following the phylogenetic‐controlled model (±95% C.I.). (A) Nucleus:Cell ratio ^a^ represents values where nucleus volumes were measured directly. See Fig. S2 for silhouette labels and individual plots and Fig. S5 for formal analysis of the allometric scaling relationships between nucleus volume and cell volume. (B) Nucleus:Cell ratio ^b^ represents values where nucleus volume was inferred from DNA content using the model in Fig. S1. See Fig. S6 for silhouette labels and individual plots, and Fig. S7 for formal analysis of the allometric scaling relationship between nucleus volume and cell volume. All slope coefficients are summarised in Fig. S3 (red symbols for Nucleus:Cell ratio ^a^ and blue symbols for Nucleus:Cell ratio ^b^).

For all but a few clades, N:C ratios decreased with increasing cell size (Fig. 1A; Fig. S2). Bacteria and birds showed the steepest decreases, whereas fish and frogs the shallowest (and not statistically significant; cf. full and empty red symbols in Fig. S3).

We derived a second, more extensive dataset on N:C ratio by converting DNA content into nucleus volume. Note that we comprehensively determined that DNA content was a robust predictor of nucleus size across species (see Materials and Methods and Fig. S1). For this more comprehensive analysis, all clades showed a decrease in N:C ratio with increasing cell size (Fig. 1B). For those clades (i.e., fish and frogs) where we could not detect a significant relationship when the nucleus was measured directly became significant using the more powerful, augmented dataset (Fig. S3). Of the nine clades included in this analysis, only phytoplankton species showed a slope in N:C ratio that slightly overlapped 0 (i.e., from −0.31 to 0.02), whereas all other species were significantly less than zero (cf. full and empty blue symbols in Fig. S3).

### N:C RATIO WITHIN SPECIES

**Figure 2:**
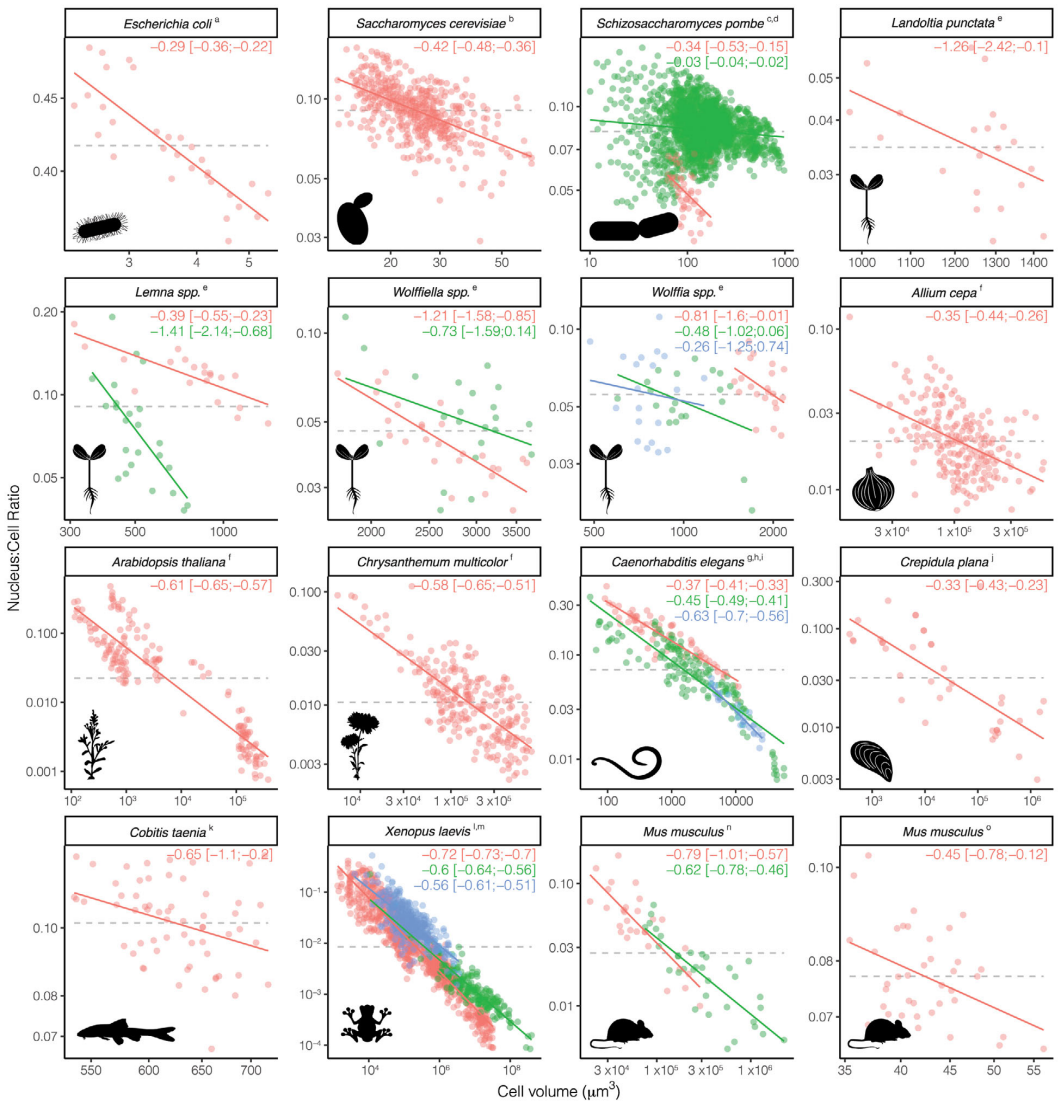
Within‐species comparison of nucleus volume to cell volume ratio and cell volume (all axes are log_10_‐transformed). Colors within each panel differentiate among different species within the same genus or among different datasets of the same species. Continues lines represent model fits whose 95% C.I. do not include 0 (i.e. 23 out of 26), with slope coefficients reported in each panel. Grey dashed lines indicate the null hypothesis of a size‐invariant N:C ratio (i.e. slope = 0 and intercept estimated from the data). Allometric slope coefficients were inferred from fitting allometric relationships between nucleus size and cell size in Fig. S8 (see Method section ‘Interpreting trends in N:C ratio across cell size’ for more details). See Fig. S4 for a summary of the slope coefficients. The superscript in the panel title indicates the reference: Gray et al. (2019) ^a^ for bacteria; Jorgensen et al. (2007) ^b^, Cantwell and Nurse (2019) ^c^, and Neuman and Nurse (2007) ^d^ for yeast; Hoang et al. (2019) ^e^ for guard cells of duckweeds; Jovtchev et al. (2006) ^f^ for mixed angiosperm leaf cells; Arata et al. (2015) ^g^, Hara et al. (2013) ^h^, and Ladouceur et al. (2015) ^i^ for nematode embryos; Conklin (1912) ^j^ for mollusc embryos; Maciak et al. (2011) ^k^ for fish embryos; Gibeaux et al. (2018) ^l^ and and Jevtic et al. (2015) ^m^ for amphibian embryos; Jaasma et al. (2006) ^n^ for mammal fibroblasts and osteoblastic cells; and Tsichlaki and FitzHarris (2016) ° for mammal embryos.

Within species, N:C ratios always decreased with cell volume (Fig. 2). The nucleus of a smaller cell occupied up to 15% of its total intracellular space, whereas the nucleus of a larger cell occupied as little as 1–2% (Fig. 2). Overall, the relative proportion of cytoplasmic space taken up by the nucleus was around 10 times greater in the smallest cells than in the largest ones. Cell size was therefore much more variable than nucleus size – a 10‐fold increase in cell volume corresponded to a 2.4‐ to 5.1‐fold increase in nucleus volume for most species (Fig. 2).

Bacteria and yeasts recorded the shallowest slopes (from −0.03 to −0.42) indicating that N:C ratios declined only slightly with increasing cell size. In contrast, mammals and frogs showed the steepest declines in N:C ratio with increasing cell size (slopes of −0.56 to −0.79 respectively). Only three species had C.I. of the slopes that overlapped 0 and all come from a single study on duckweed species with relatively low sample sizes (N = 20–25 per species) and the highest uncertainties (Fig. S4).

### COEVOLUTION OF CELL AND NUCLEUS SIZE

Trials for this experiment took place after 350 and 450 generations of artificially selecting the eukaryotic microalga *Dunaliella tertiolecta* for cell size, when mean cell volumes were on average 97 μm^3^ for small‐selected lineages, 177 μm^3^ for control, and 915 μm^3^ for large‐selected lineages. Hence, large‐selected cells were on average 9.4 times larger than small‐selected cells and this difference was comparable across trials.

**Figure 3:**
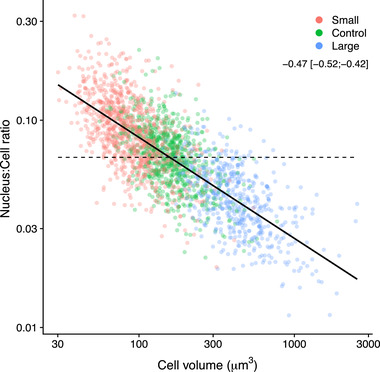
Nucleus volume to cell volume ratio (N:C ratio) as a function of cell volume among cells of *Dunaliella tertiolecta* that were artificial selected for size (both axes are log_10_‐transformed). Each point is a cell after correcting for blocking factor (i.e. generation time) and random covariates (i.e. slope and intercept for the lineage identity, nested within generation). The color of the points represents the size‐selection treatment. Continuous line shows the fit of a linear mixed‐effect model, whose slope coefficient is reported in the legend [±95% C.I.]. Dashed line displays the null hypothesis of a size‐invariant N:C ratio (i.e. slope = 0 and intercept estimated from the data). The allometric slope coefficient was inferred from fitting an allometric regression between nucleus volume and cell volume in Fig. S9 (see Method section ‘Interpreting trends in N:C ratio across cell size’ for more details).

Two separate experiments showed that N:C ratios declined with cell size (Fig. 3). As cells evolved to larger sizes, the ratio of nucleus volume to cell volume decreased from ∼7‐8% of the total volume of a 100 μm^3^ cell, to only ∼2‐3% of a 1000 μm^3^ cell (Fig. 3).

## Discussion

We showed that larger cells have relatively smaller nuclei at all scales of biological organization across the Tree of Life: among‐species, within‐species, and among‐lineages of a species that was artificially selected for cell size. While larger cells typically had *absolutely* larger nuclei, the ratio of nucleus to cell (N:C) size always decreased with cell size.

A negative relationship between N:C ratios and cell size has the corollary that cells vary in size much more than nuclei across the Tree of Life. Cell size varied across six orders of magnitude while nucleus size varied by only three orders of magnitude. Whether this pattern reflects stronger stabilizing selection on nucleus size or stronger disruptive selection on cell size is unclear. What is clear is that when cells are larger, nuclei are relatively smaller. Our analysis across lineages of the green alga *Dunaliella tertiolecta* implies that this pattern may be the product of selection. When we experimentally evolved cells to be of very different sizes, nucleus size evolved much less. These results and patterns across‐ and within‐species more generally (Niklas 2015; Niklas and Hammond 2019) suggest that rather than a single N:C ratio being optimal, as has been argued in the past (Cavalier‐Smith 2005), selection favors different N:C ratios, depending on absolute cell sizes.

Why do larger cells have relatively smaller nuclei? In eukaryotes, cellular metabolism scales hypo‐allometrically with cell size – in other words, larger cells also have relatively lower metabolisms (Gregory 2002; West *et al*. 2002; Kozlowski *et al*. 2003). It is intuitively appealing to assume that larger cells, with their lower relative metabolic rates might therefore be able to meet all of their functions with relatively smaller nuclei. However, the reverse could also hold: it is easy to imagine larger cells, with relatively smaller nuclei only being capable of sustaining relatively lower metabolic rates. Whether hypoallometric scaling of metabolism drives hypoallometric scaling of nucleus size, or *vice versa*, remains unclear. A first step to determine causality would be to experimentally manipulate the N:C ratio (as per Jorgensen *et al*. (2007) and Neumann and Nurse (2007)) and then estimate metabolic scaling.

Some of our within‐species datasets for multicellular organisms included functionally diverse cells (e.g., mixed epithelial cells, amphibian embryos, nematode eggs). In these cases, cells of different sizes may have different functions, which could influence the shape of the relationship between cell volume and nucleus volume. Yet, within‐species datasets of functionally equivalent unicellular species showed decreasing trends in N:C ratios that were similar to heterogeneous cells of functionally diverse multicellular species. Moreover, all among‐species datasets of multicellular organisms included in this study were from functionally similar cells (i.e., red blood cells) and they also showed a comparable decrease in N:C with cell size. Hence, cell functionality is unlikely to play an important role in explaining the systematic decrease of N:C ratio with cell volume.

Regardless of how we measured genome size (nucleus size or DNA content), both measures show that N:C ratios declined with cell size, but our results differed slightly between measures. When estimated using the size of the nucleus (or of the nucleoid), the relationship between N:C ratio and cell size is remarkably consistent across diverse groups, from bacteria to birds, and scales with an exponent of −0.3 across 5 orders of magnitude. In contrast, the decline in N:C ratio estimated using DNA content was much more taxon‐specific; some groups recording relatively shallow declines (phytoplankton), while endotherms (birds and mammals) showed extremely steep declines.

It is intriguing that only in warm‐blooded animals we find that relative DNA content declines sharply with cell size. Endotherms have systematically higher metabolic rates than ectotherms. If metabolism and N:C ratios are linked, as others have suggested (Vinogradov 1997; Gregory 2002; Kozlowski *et al*. 2003; Maciak *et al*. 2011), then this may explain why we observe systematic differences between endotherms and ectotherms. However, at this stage, we are reluctant to attribute the striking pattern in mammals and birds solely to endothermy.

Our findings emphasize the need for quantitative theory regarding the scaling of N:C ratios. Most N:C hypotheses make qualitative predictions of a positive relationship between cell size and nucleus size. We find such a positive relationship (for the most part), but quantitative theory regarding the precise shape of the positive relationship is scarce (but see Niklas 2015; Niklas and Hammond 2019). One potentially fruitful approach would be to apply the metabolic model in Kozlowski and Weiner (1997). This model was developed to understand how cell size and genome size affect variation in metabolic rate but might be re‐arranged to explore how metabolic rate affects the N:C ratio. Of course, the relationship between nucleus size and cell size becomes more complex in multicellular organisms with complex cellular architectures. In such cases, the N:C ratio may change across hierarchical levels of organization (i.e., cells, tissues, organs, whole organism) and also between metabolically inert organs (e.g., skeleton, hair) and metabolically active organs (e.g., heart, muscles). Hence, another useful approach may be to investigate trends between N:C ratio and cell‐specific metabolic rate among tissues of the same organism (Kozlowski *et al*. 2020).

In conclusion, we see strong evidence for a decrease in the ratio of nucleus size to cell size in every domain of life that we explored: larger cells almost invariably have relatively smaller nuclei. While cell size and nucleus size are highly variable in nature, the remarkable consistency of a decrease in relative nucleus size in larger cells provides hope that some universal driver of this relationship might one day be identified.

## Methods

### META‐ANALYSIS

We carried out two meta‐analyses on the ratio between nucleus volume and cell volume (N:C ratio) among cells, one within‐species and another among‐species. Together with data on eukaryotic cells, we also included data on the nucleoid volume of bacteria.

#### Among species

We compiled a dataset on N:C ratio among species from the Animal Genome Size project (http://www.genomesize.com/cellsize), an open‐source database gathered from the scientific literature (Gregory *et al*. 2007; Gregory 2019) reporting cell volume and nucleus volume (both μm^3^) for red blood cells (erythrocyte) among species of fish (N = 43), birds (N = 105), reptiles (N = 38), and amphibians (N = 73, after complementing with Wei *et al*. 2015). We also included data for the nucleoid – the analogue of the nucleus in bacteria (N = 37) – that were sourced from Gray *et al*. (2019).

Studies report the DNA content of a cell more frequently than the nucleus size and the two are typically proportional (Cavalier‐Smith 2005; Jovtchev *et al*. 2006). So, we compiled a second, independent dataset where we inferred nucleus volume from DNA content. We sourced data from the Animal Genome Size project (http://www.genomesize.com/), which reports cell DNA content (pg) and cell volume for red blood cells of birds (N = 183), mammals (N = 116), reptiles (N = 38), and amphibians (N = 65). In addition, we sourced data for phytoplankton (N = 49) from Beaton and Cavalier‐Smith (1999), LaJeunesse *et al*. (2005), and Shuter *et al*. (1983); for prokaryotes (N = 18) from Shuter *et al*. (1983); and for blood cells of fish (N = 198) from Hardie and Hebert (2003). We developed a calibration curve to convert DNA content (pg) to nucleus volume (μm^3^) using 178 species across five clades for which we had both information (Fig. S1A). All clades showed a statistically consistent slope between DNA content and nucleus volume (i.e., interaction between DNA content and taxonomic clade was not statistically significant). Therefore, we used a calibration curve with a single slope and clade‐specific intercepts (Fig. S1A). Model predictions were precise (*R* ^2^ = 0.94) and showed comparable levels of uncertainty among clades, with fish being the most precise and aves the least (see Fig. S1B). Overall, the nearly perfectly isometric size‐scaling exponent (1.03) implies that the slope between DNA content and cell size was equivalent to the slope between DNA‐inferred nucleus size and cell size. Hence, the overall conclusions were unaffected by including DNA‐inferred nucleus sizes, except that they added statistical power to our tests.

Traits of taxonomically related species may be correlated and not statistically independent. Hence, phylogenetic similarities across species need to be incorporated in the variance structure to avoid violating the assumptions of most statistical tests (Lynch 1991; Housworth *et al*. 2004). We fitted two separate phylogenetic mixed models to assess the relationships between nucleus volume and cell volume: one where nucleus volume was directly measured, and another where nucleus volume was inferred from DNA content using a calibration curve. In both models, we fitted Bayesian phylogenetic mixed‐models using the Metropolis‐Hasting sampler (Hadfield 2010) in the R package MCMCglmm. The response variable was the volume of the nucleus. Fixed covariates were cell volume (continuous), phylum (discrete), and their interaction. If the credible intervals for the interaction coefficient overlapped 0, the model was re‐fitted including only main effects. The phylogeny of the species was included in the variance structure of among‐species models as a random effect (parameter “random”) and was compiled from the Tree of Life Web Project (Maddison *et al*. 2007), using the R package rotl (Michonneau *et al*. 2016). We divided the phylum Amphibia into frogs and salamanders because these groups showed non‐overlapping covariate ranges. We also added clade as a residual covariance structure for the six taxonomic clades (parameter “rcov”). All priors were uninformative from an inverse Wishart distribution. We used 500,000 iterations (parameter “nitt”), thinning every 100 iterations (parameter “thin”), and a burn‐in of 10,000 (parameter “burnin”). When more than one value was reported for a species, we only included the average in the analyses. To monitor successful convergence, we ran multiple chains, inspected the iterated history, density plot, and ensured Geweke *z*‐score between −2 and 2 (Geweke 1992).

#### Within species

We compiled a within‐species dataset on N:C ratios from digitalising plots of scientific articles monitoring cell size and nucleus size from populations of the same species – including wild‐types and mutants. We only included studies with at least 20 observations. The species represented in this dataset were: the fission yeast (Neumann and Nurse 2007; Cantwell and Nurse 2019), the budding yeast (Jorgensen *et al*. 2007), the blastomere of a marine gastropod (Conklin 1912), *Escherichia coli* (Gray *et al*. 2019), eggs of a nematode (Hara *et al*. 2013; Arata *et al*. 2015; Ladouceur *et al*. 2015), mixed epidermal cells from leaves of three angiosperm species (Jovtchev *et al*. 2006), guard cells of eight duckweed species (Hoang *et al*. 2019), red blood cells of a fish (Maciak *et al*. 2011), embryos of an amphibian (Jevtic and Levy 2015; Gibeaux *et al*. 2018), and fibroblasts and osteoblastic cells cells and embryos of a mammal (Jaasma *et al*. 2006; Tsichlaki and FitzHarris 2016) (see Fig. 2 or Fig. S4 for species names). Overall, our within‐species dataset covers both unicellular organisms and multicellular organisms (which we standardized for equivalent cell type). Also, we excluded strains of fission yeast in Cantwell and Nurse (2019) whose N:C ratio was experimentally manipulated. We converted data reported in other size units to cell volume (μm^3^) using the formulas $\frac{4}{3}\pi {\left(\sqrt{\frac{Area}{\pi }}\right)}^{3}$ or $\frac{4}{3}\pi {\left(\frac{Diameter}{2}\right)}^{3}$.

Various statistical techniques have been used to analyze allometric relationships and there is debate about which is most appropriate (Legendre and Legendre 2012). Here we followed the recommendation by Kilmer and Rodriguez (2017) to use ordinary least‐square regressions for allometric studies in evolutionary biology. Importantly, we verified that all conclusions remained unaffected when using major axis regressions or quantile regressions (analyses not shown). We analyzed each dataset by fitting ordinary least‐square regressions on log_10_‐log_10_ data between nucleus volume (dependent variable) and cell volume (explanatory variable). The N:C ratio was deemed to be size‐invariant if the slope coefficient included 1 in its 95% confidence interval.

### ARTIFICIAL SELECTION FOR CELL SIZE AND THE REPERCUSSIONS ON THE NUCLEUS

#### Long‐term artificial selection program

We used 3 years of artificial selection to evolve an ancestral population of a unicellular green microalga (*Dunaliella tertiolecta*) to different cell sizes, while controlling for the influence of other biotic and abiotic factors. For details on the artificial selection protocols, refer to Malerba *et al*. (2018). Briefly, we sourced the cosmopolitan, fast‐growing green microalgal species *Dunaliella tertiolecta* (Butcher) from the Australian National Algae Culture Collection (ANACC; strain code CS‐14). We kept mother cultures in a temperature‐controlled room at 21 ± 1°C, reared in autoclaved F/2 medium (no silica) from 0.45 μm‐filtered seawater (Guillard 1975). Light intensity was at 150 μM photos m^−2^ s^−1^ with a photoperiod of 14‐10 day‐night cycle, using low‐heat 50 W LED flood lights (Power‐lite^TM^, Nedlands Group, Bedfordale, Australia). The artificial selection methods rely on larger cells forming a pellet at the bottom of test tubes at lower centrifugal forces compared to smaller cells, which instead will remain in solution (i.e., differential centrifugation). On April 25, 2016, we inoculated 72 lineages using the same ancestral population of *D. tertiolecta* into aseptic 75 cm^2^ plastic cell culture flasks (Corning, Canted Neck, Nonpyrogenic). Since then, lineages have been selected twice a week, each Monday and Thursday: 30 lineages were large‐selected, 30 small‐selected, and 12 were the control. Control cultures experienced identical conditions (including centrifugation) without being size‐selected. At the end of selection, all cultures were reinoculated into fresh F/2 medium.

#### Cell volume and nucleus volume

For each of the three artificial selection treatments, we randomly sampled 12 lineages after 350 and 450 generations (total of 72 lineages). To remove any environmental effects and non‐genetic phenotypic differences from artificial selection, we grew cells for three generations (a week) under common garden conditions with no centrifugation (neutral selection) before starting any trial.

Following neutral selection, we measured the mean cell volume of all sampled lineages, using optic light microscopy at 400x after staining cells with lugol's iodine at 2%. We calculated cell volume from around 200 cells per culture in Fiji 2.0 (Schindelin *et al*. 2012) assuming prolate spheroid shape, as recommended for this species by Hillebrand *et al*. (1999).

We first fixed samples in 2% glutaraldehyde and then resuspended them into growth medium. We diluted fixed samples to approximately 3 × 10^6^ cells/mL and stained them with DAPI (4′,6‐diamidino‐2‐phenylindole) to attain a final dye concentration of 0.1 μg/mL. This dye can penetrate fixed cells and bind with DNA to form a fluorescence with an absorption maximum at 358 nm (ultraviolet) and an emission maximum at 461 nm (blue). We incubated stained cells in the dark for 30 minutes and imaged them on a slide with a fluorescent inverted microscope (Leica DMi8). For each lineage, we took 20 photos with both brightfield view for cell size and with the DAPI channel (excitation = 325–375nm; emission = 435–485nm) for nucleus size. Nucleus volume was calculated with Fiji 2.0 by assuming a prolate spheroidal shape. The linear mixed‐effect model to calculate the size‐scaling coefficient between cell volume and nucleus volume included a fixed blocking variable describing whether the culture was measured after 350 (N = 1332) or 450 (N = 1058) generations of artificial selection and a random slope and a random intercept for each lineage nested within generation.

We carried out all analyses in this study using R (R Core Team 2019) and the packages nlme (Pinheiro *et al*. 2016), lme4 (Bates *et al*. 2015), and MCMCglmm (Hadfield 2010) for model fitting and ggplot2 (Wickham 2009) and cowplot (Wilke 2016) for plotting.

### INTERPRETING TRENDS IN N:C RATIO ACROSS CELL SIZE

Regressing N:C ratio with cell size can produce spurious results, because cell size is both the explanatory variable and the denominator of the response variable, voiding the assumption of independency in linear models (Brett 2004). Therefore, we evaluated how nucleus size changed with cell size by fitting allometric scaling relationships of the form $nucleus\;volume\;=\;a\times \;cell\;volume^{b}$, where a is the normalization constant and b is the size‐scaling exponent.

All allometric slope coefficients (b) and 95% C. I. were estimated from regressing nucleus size (dependent variable) with cell size (explanatory variable). There was no model fitting based on N:C data. Yet, to more easily visualize trends in N:C ratios, we rearranged the calibrated models by dividing both sides by cell size, as $\frac{nucleus\;volume}{cell\;volume}=\;a\times \;\frac{cell\;volume^{b}}{cell\;volume}$, which became $\frac{nucleus\;volume}{cell\;volume}\;=\;a\times cell\;volume^{b-1}$ and $\mathrm{lo}g_{10}\left(\frac{\mathrm{nucl}\mathrm{eus}\;\mathrm{volu}\mathrm{me}}{\mathrm{cell}\;\mathrm{volu}\mathrm{me}}\right)=\mathrm{lo}g_{10}\left(a\right)+\;\left(b-1\right)\times \mathrm{lo}g_{10}\left(\mathrm{cell}\;\mathrm{volu}\mathrm{me}\right)$. In this form, the slope of the allometric relationship became b−1. If the slope coefficient (b−1) included 0 in its 95% confidence interval, the relationship between cell size and nucleus size was deemed isometric (i.e., doubling cell size corresponds to doubling nucleus size), which implied a constant N:C ratio. Conversely, b−1 statistically greater than 1 indicated N:C ratios increasing with cell size (hyper‐allometry), and b−1 statistically lower than 1 implied N:C ratios decreasing with cell size (hypo‐allometry). Importantly, our methods to rearrange the equation *after* the slope coefficient (*b*) was already calibrated preserved the independence of our response (nucleus size) and predictor (cell size) variables.

## AUTHOR CONTRIBUTIONS

Both authors contributed to designing the study. M.E.M. conducted the experiment and collected the data. M.E.M. and D.J.M. carried out statistical analyses and wrote the initial draft of the manuscript. Both authors gave final approval for publication.

## DATA ARCHIVING

All data generated in this study are available in Dryad (https://doi.org/10.5061/dryad.vq83bk3ss).

## Supporting information


**Figure S1**: (A) Relationship between nucleus volume and cell DNA content among clades (R^2^ = 0.94). Axes are log_10_‐transformed.
**Figure S2**: Nucleus volume to cell volume ratio as a function of cell volume across species of different clades (all axes are log_10_‐transformed).
**Figure S3**: Slope coefficients (±95% C.I.) of linear models between log_10_(nucleus:cell) and log_10_(cell size) across species of different clades.
**Figure S4**: Slope coefficients (±95% C.I.) of linear models between log_10_(nucleus:cell) and log_10_(cell size) for each dataset.
**Figure S5**: Nucleus volumes and cell volumes across species of different clades (all axes are log_10_‐transformed).
**Figure S6**: Nucleus volume to cell volume ratio as a function of cell volume among species of different clades (all axes are log_10_‐transfromed).
**Figure S7**: Nucleus volumes and cell volumes among species of different clades (both axes are log_10_‐transfromed).
**Figure S8**: Nucleus volume and cell volume for cells within the same species (both axes are log_10_‐transfromed).
**Figure S9**: Nucleus volume and cell volume among cells of *Dunaliella tertiolecta* that were artificial selected for size (both axes are log_10_‐transfromed).
