# Supplementary material for: Larger cells have relatively smaller nuclei across the Tree of Life
Source: Evol Lett. 2021 Jun 29;5(4):306–14. doi: 10.1002/evl3.243 (PMC8327945; doi:10.1002/evl3.243)
Supplement: Supplementary file 1 — Figure S1: (A) Relationship between nucleus volume and cell DNA content among clades (R2 = 0.94). Axes are log10‐transformed. Figure S2: Nucleus volume to cell volume ratio as a function of cell volume across species of different clades (all axes are log10‐transformed). Figure S3: Slope coefficients (±95% C.I.) of linear models between log10(nucleus:cell) and log10(cell size) across species of different clades. Figure S4: Slope coefficients (±95% C.I.) of linear models between log10(nucleus:cell) and log10(cell size) for each dataset. Figure S5: Nucleus volumes and cell volumes across species of different clades (all axes are log10‐transformed). Figure S6: Nucleus volume to cell volume ratio as a function of cell volume among species of different clades (all axes are log10‐transfromed). Figure S7: Nucleus volumes and cell volumes among species of different clades (both axes are log10‐transfromed). Figure S8: Nucleus volume and cell volume for cells within the same species (both axes are log10‐transfromed). Figure S9: Nucleus volume and cell volume among cells of Dunaliella tertiolecta that were artificial selected for size (both axes are log10‐transfromed). [file EVL3-5-306-s001.docx]

**Supplementary information**


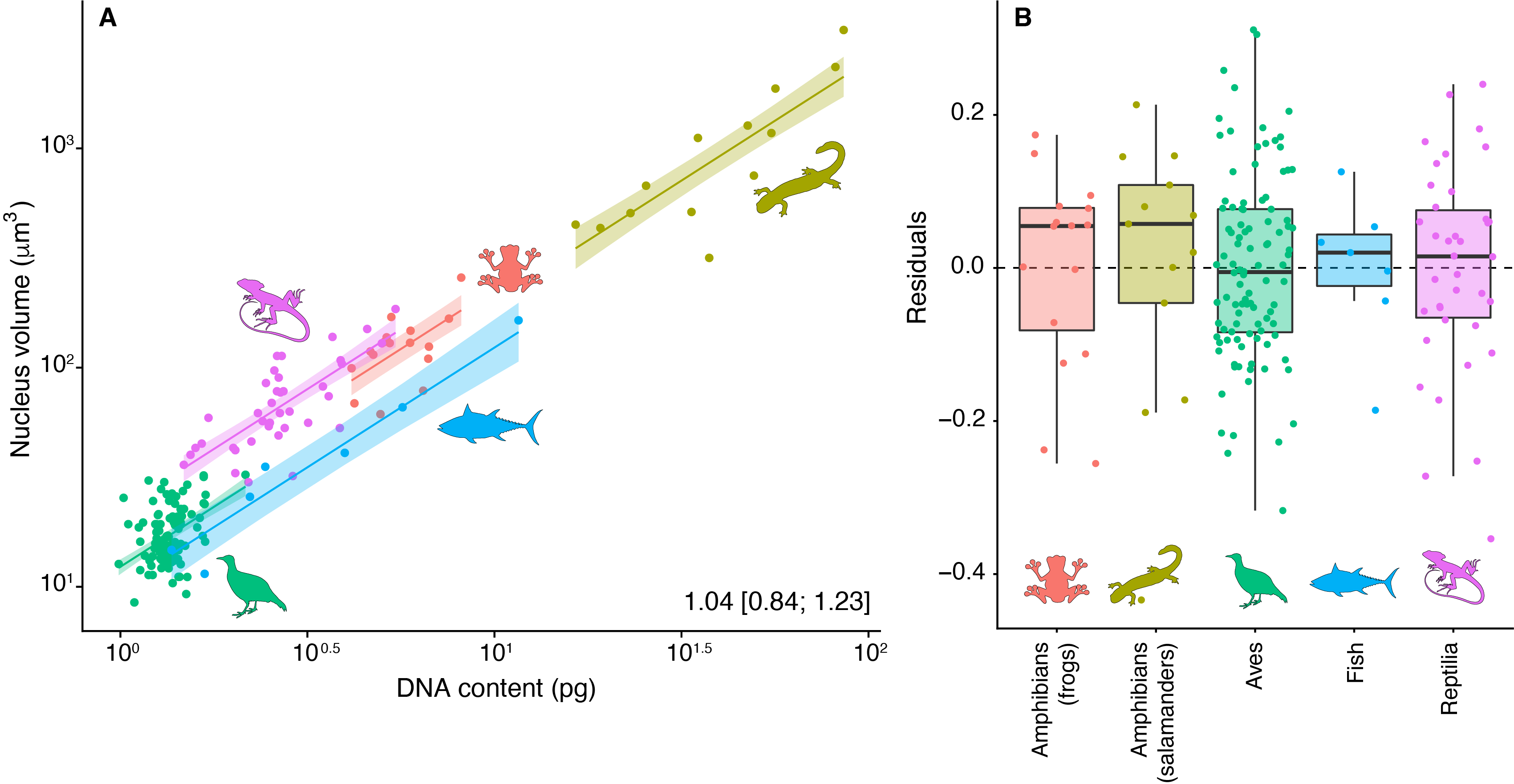


**Figure S1**: (A) Relationship between nucleus volume and cell DNA content among clades (R^2^ = 0.94). Axes are log_10_-transformed. Each point represents a species (N = 178), colour-coded for taxonomic clade. Continues lines ($\pm$95% C.I) represent the best-fitting phylogenetic-controlled model that was used to estimate nucleus volume from DNA content where direct observations were not available. The interaction between DNA content and taxonomic clade overlapped 0 and was omitted from the final model. Hence, the calibration curve between DNA content and nucleus volume consisted of a single slope and clade-specific intercepts. (B) Distribution of the residuals from the scatterplot for each clade, with each species presented as a point (with jittered x-axis position). When converting species from clades other than those reported here, we used the overall intercept across all data.

**
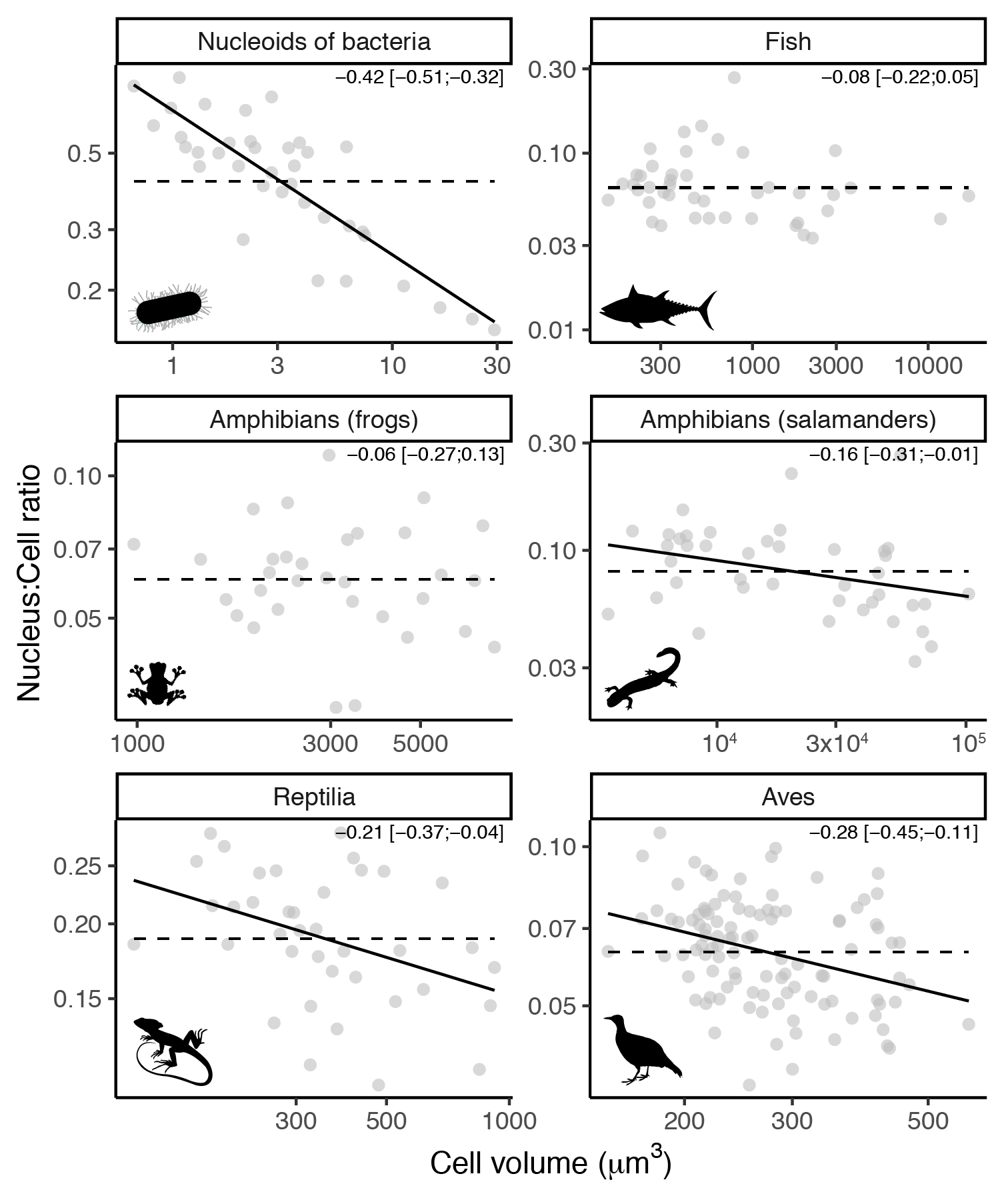
**

**Figure S2**: Nucleus volume to cell volume ratio as a function of cell volume across species of different clades (all axes are log_10_-transformed). Each dot represents a species whose nucleus size and cell size were reported in the literature. Continues lines represent the model fit of a phylogenetic mixed-model (coefficients reported on each panel [$\pm$95% C.I]), while dashed lines indicate the null hypothesis of a size-invariant N:C ratio (i.e. slope = 0 and intercept estimated from the data). These are the same relationships displayed in Fig. 1A. Allometric slope coefficients were inferred from fitting allometric relationships in Fig. S5 (see Method section ‘Interpreting trends in N:C ratio across cell size’ for more details). Notice that for prokaryotic bacteria we report the volume of the nucleoid, whereas for all other clades we report the volume of the nucleus.


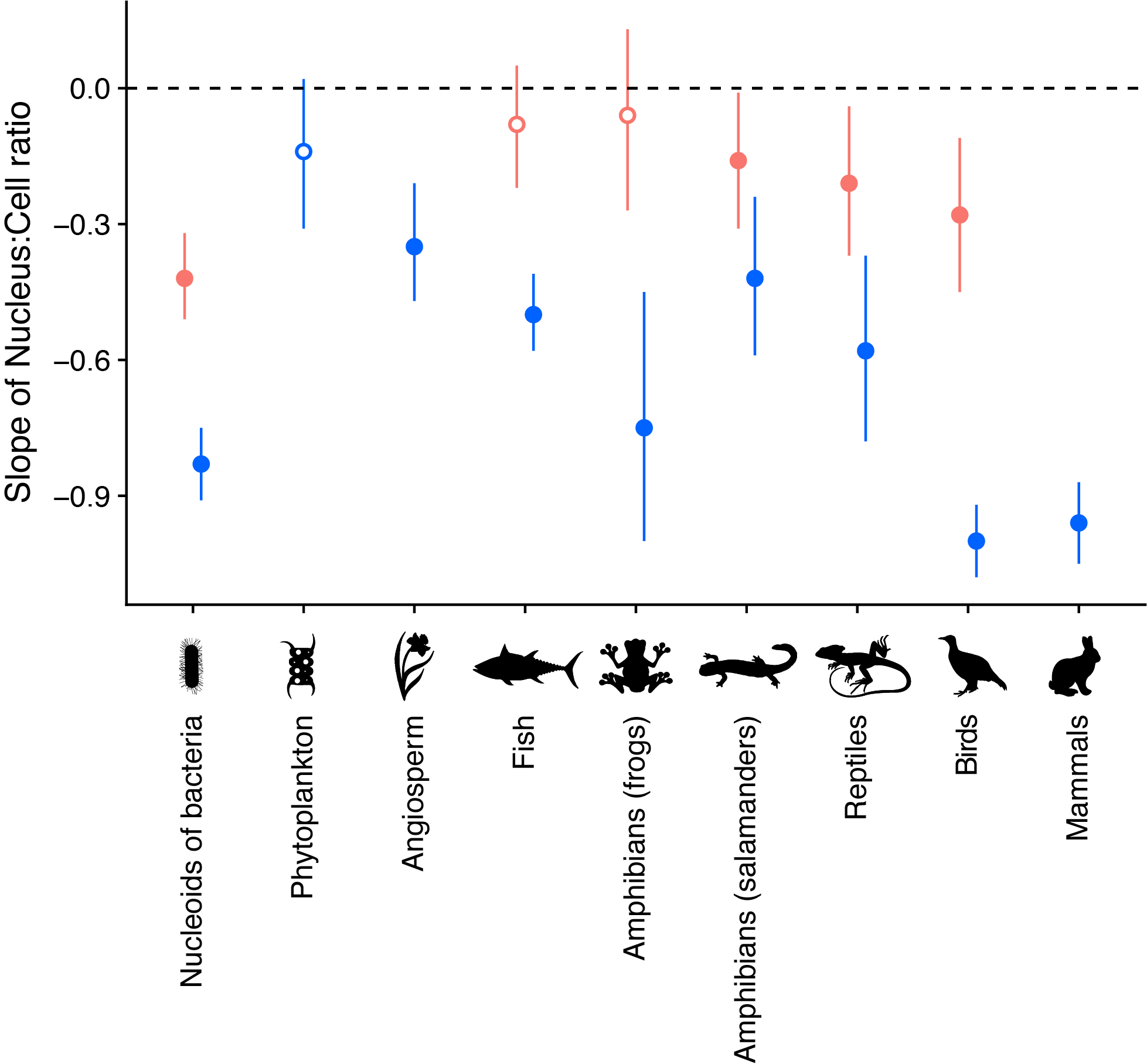


**Figure S3:** Slope coefficients ($\pm$95% C.I.) of linear models between log_10_(nucleus:cell) and log_10_(cell size) across species of different clades. Full dots indicate means whose 95% C.I. do not include 0 (i.e. 12 out of 15), which indicates a decrease in N:C ratio with increasing cell volume. Nucleus volume was either directly measured (red symbols) or was inferred from DNA content using the model in Fig. S1 (blue symbols). Allometric slope coefficients were inferred from fitting allometric relationships in Fig. S5 (red) and Fig S7 (blue; see Method section ‘Interpreting trends in N:C ratio across cell size’ for more details). See legend in Fig. 1 for raw data and more information.


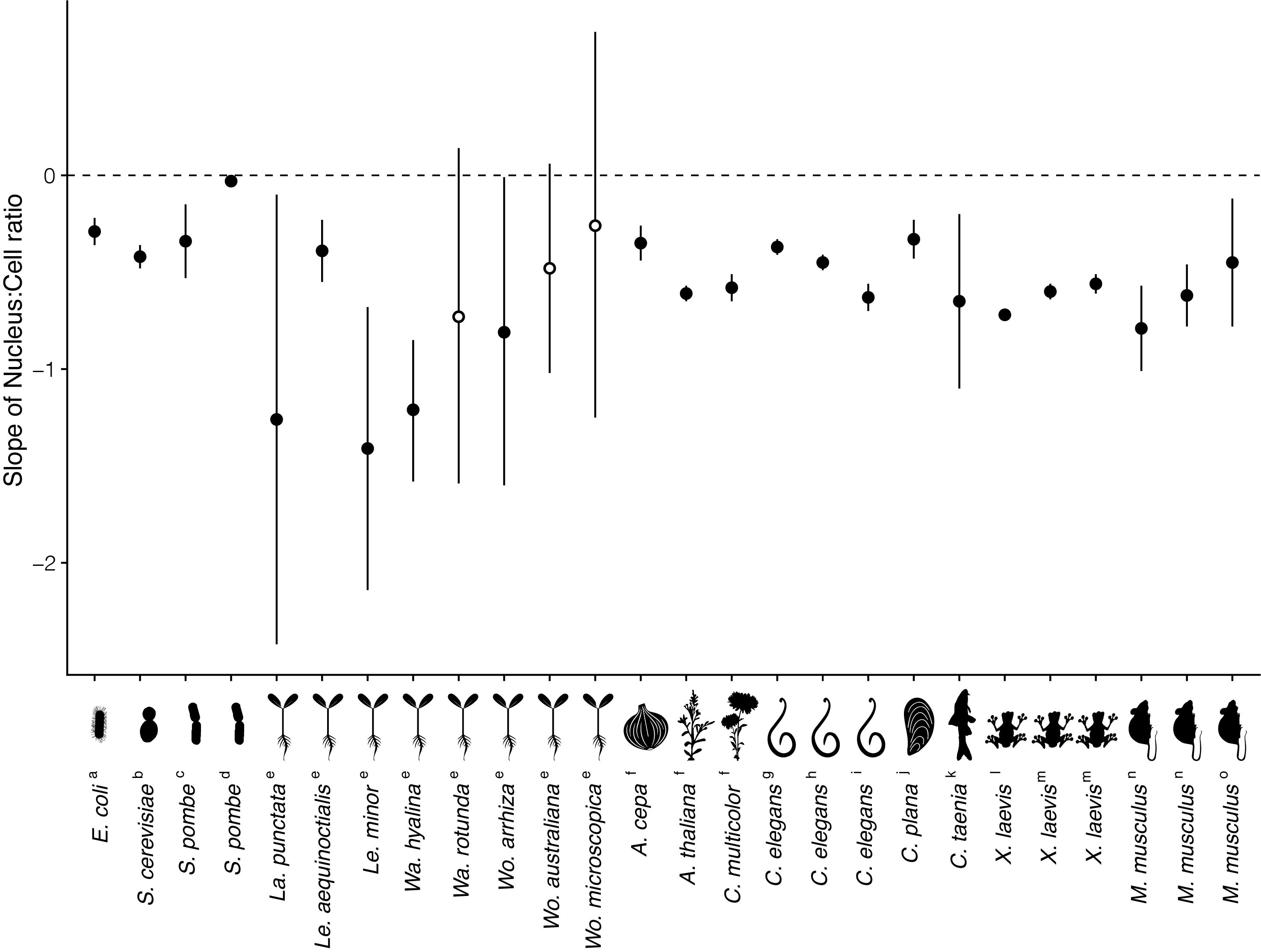


**Figure S4:** Slope coefficients ($\pm$95% C.I.) of linear models between log_10_(nucleus:cell) and log_10_(cell size) for each dataset. Full dots indicate means whose 95% C.I. do not include 0 (i.e. 23 out of 26), which indicate a decrease in N:C ratio with increasing cell volume. See legend in Fig. 2 for raw data and more information. Allometric slope coefficients were inferred from fitting allometric relationships in Fig. S8 (see Method section ‘Interpreting trends in N:C ratio across cell size’ for more details).


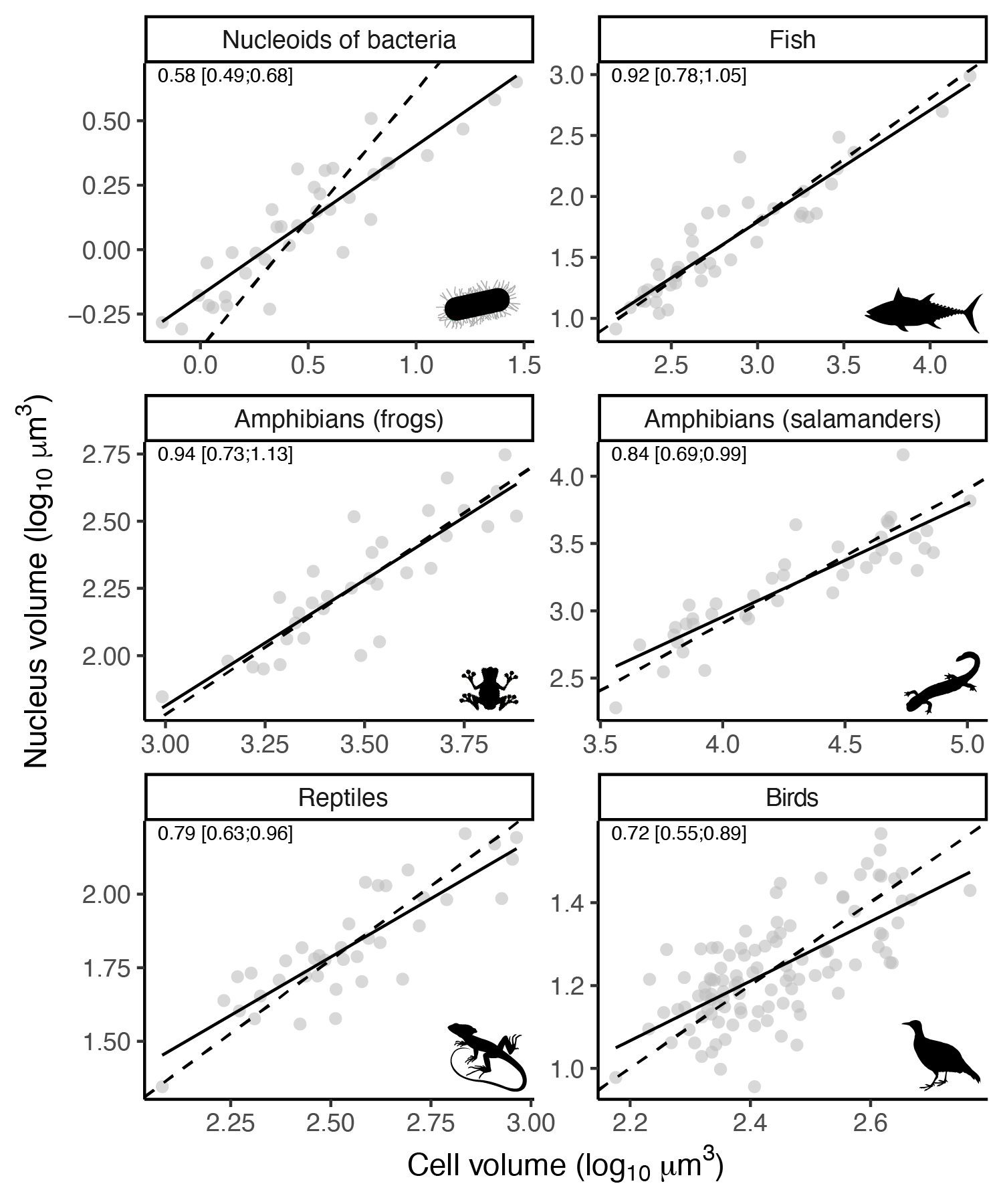


**Figure S5:** Nucleus volumes and cell volumes across species of different clades (all axes are log_10_-transformed). Each dot represents a species whose nucleus size and cell size were reported in the literature. Continues lines represent model fits of a phylogenetic mixed-model (coefficient reported on each panel [$\pm$95% C.I]), while dashed lines indicate the null hypothesis of a size-invariant N:C ratio (i.e. slope = 1 and intercept estimated from the data). These relationships are calculated on the same dataset of Fig. 1A and Fig. S2. Notice that for prokaryotic bacteria we report the volume of the nucleoid, whereas for all other clades we report the volume of the nucleus.


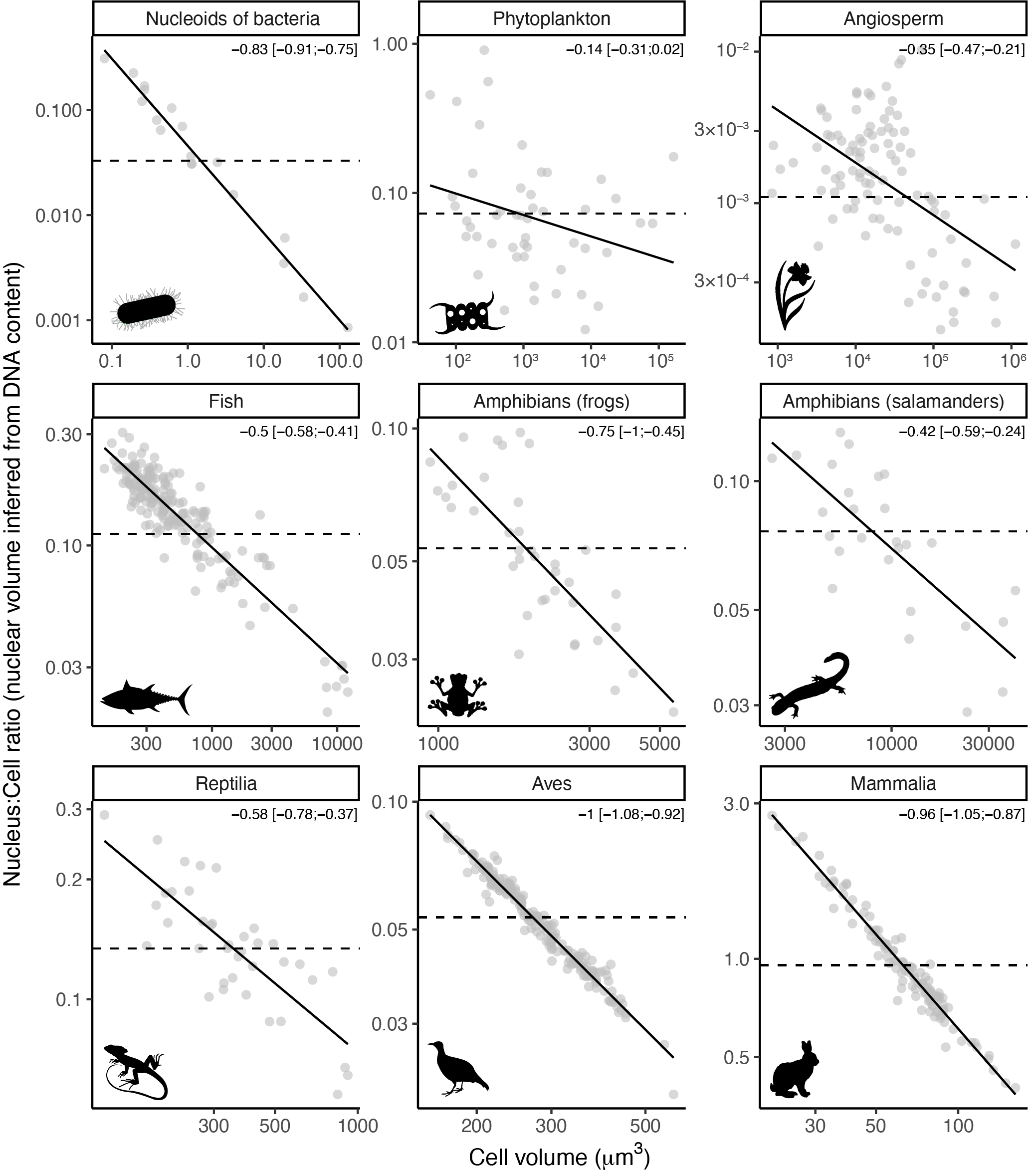


**Figure S6:** Nucleus volume to cell volume ratio as a function of cell volume among species of different clades (all axes are log_10_-transfromed). Each dot represents a species whose cell size and DNA content were reported in the literature. Nucleus volume was inferred from DNA content using the model in Fig. S1. Continues lines represent model fits of a phylogenetic mixed-model (coefficient reported on each panel [$\pm$95% C.I]), while dashed lines indicate the null hypothesis of a size-invariant N:C ratio (i.e. slope = 0 and intercept estimated from the data). These data and model fits are identical to Fig. 1B. Slope coefficients were inferred from fitting the allometric relationships in Fig. S7 (see Method section ‘Interpreting trends in N:C ratio across cell size’ for more details). Notice that for prokaryotic bacteria we report the volume of the nucleoid, whereas for all other clades we report the volume of the nucleus.


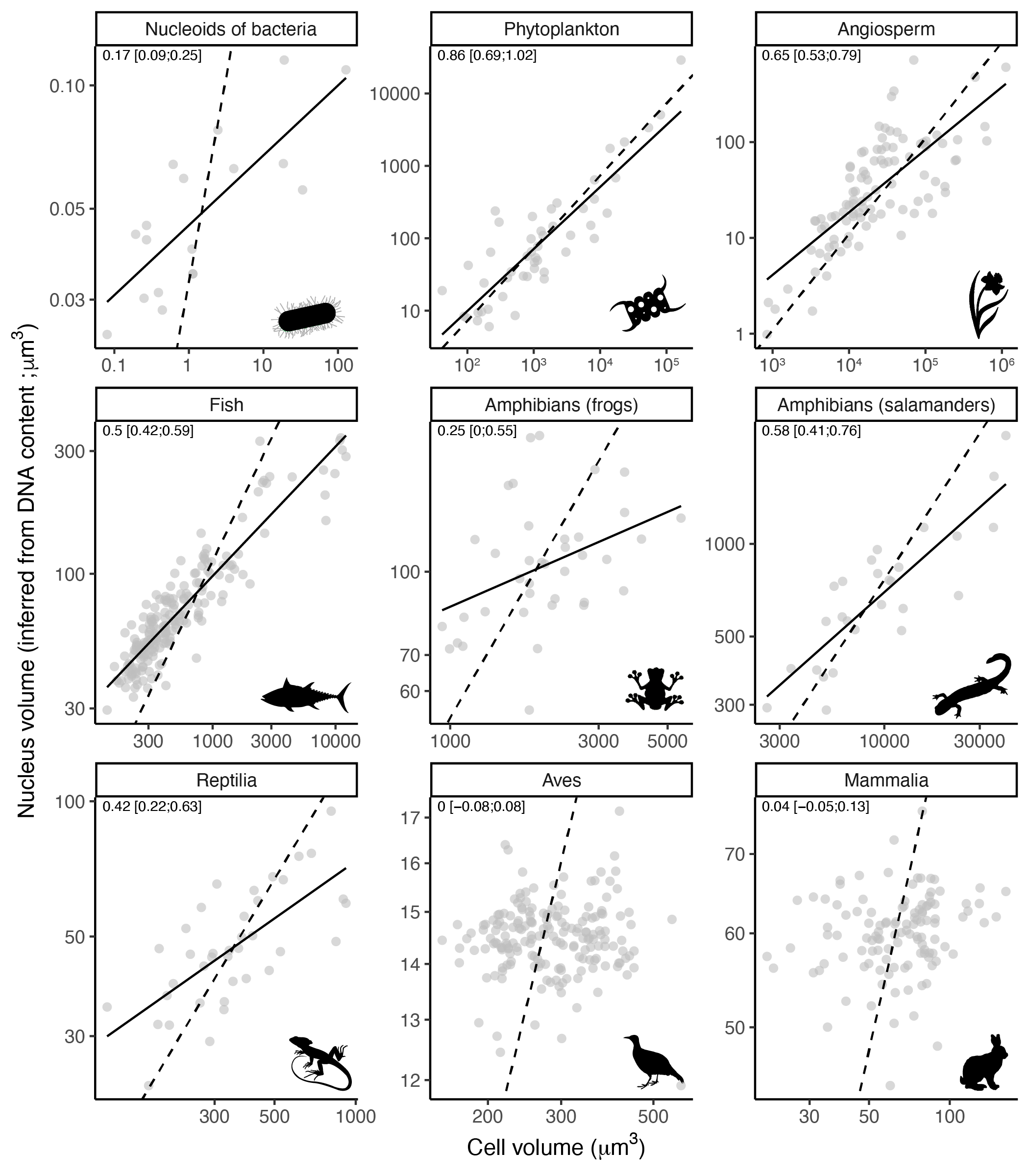


**Figure S7:** Nucleus volumes and cell volumes among species of different clades (both axes are log_10_-transfromed). Each dot represents a species whose cell size and DNA content were reported in the literature. Nucleus volume was inferred from DNA content using the model in Fig. S1. Continues lines represent the model fit of a phylogenetic mixed-model (coefficients reported on each panel [$\pm$95% C.I]), while dashed lines indicate the null hypothesis of a size-invariant N:C ratio (i.e. slope = 1 and intercept estimated from the data). These relationships are calculated on the same dataset of Fig. 1B and Fig. S6. Notice that for prokaryotic bacteria we report the volume of the nucleoid, whereas for all other clades we report the volume of the nucleus.


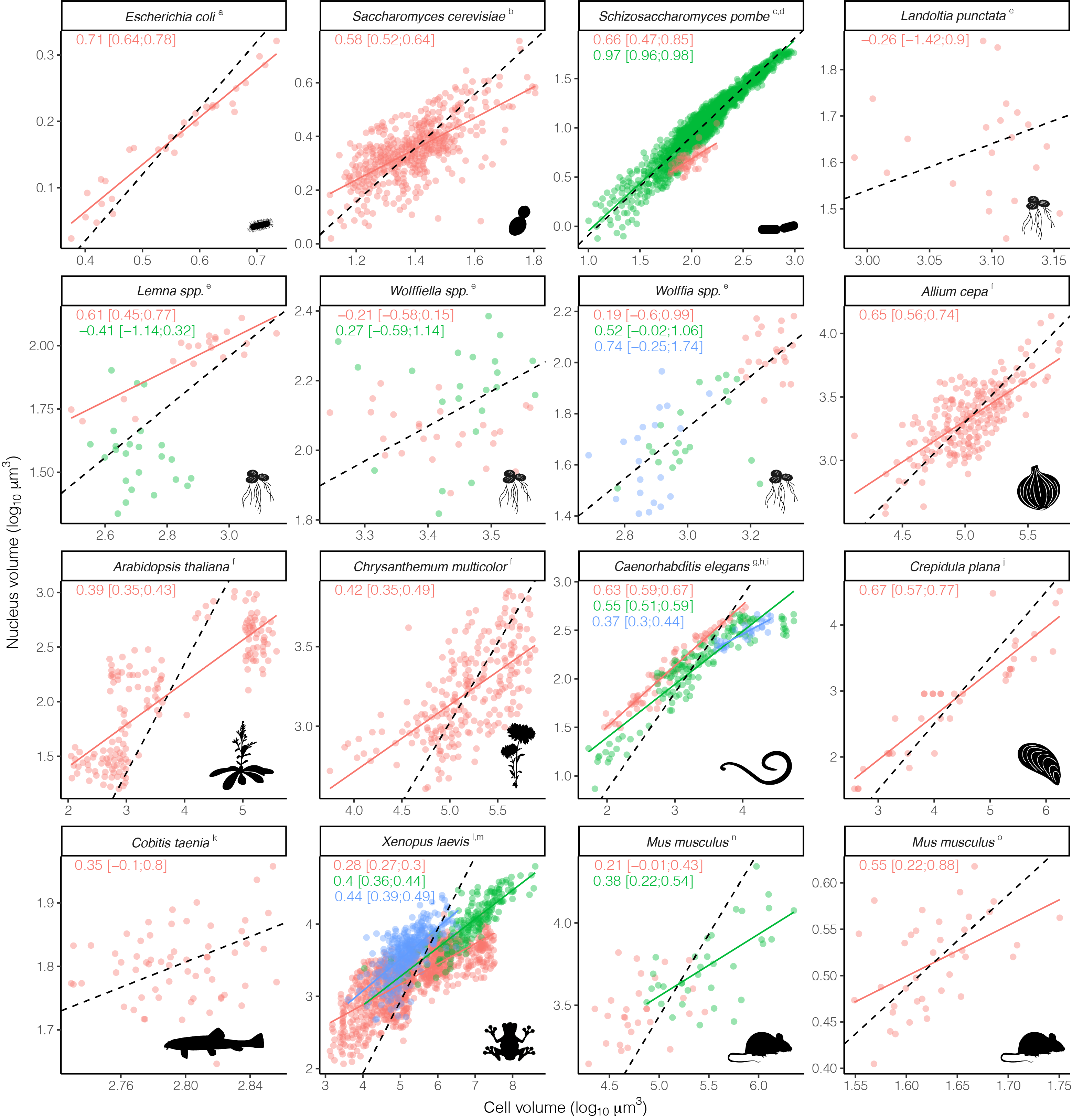


**Figure S8:** Nucleus volume and cell volume for cells within the same species (both axes are log_10_-transfromed). Colours within each panel differentiate among different species within the same genus or among different datasets of the same species. Continues lines represent model fits whose 95% C.I. do not include 0 (i.e. 17 out of 26), with slope coefficients reported in each panel. Grey dashed lines indicate the null hypothesis of a size-invariant N:C ratio (i.e. slope = 0 and intercept estimated from the data). The superscript in the panel tittle indicates the source of the data: Gray et al. (2019) ^a^, Jorgensen et al. (2007) ^b^, Cantwell and Nurse (2019) ^c^, Neuman and Nurse (2007) ^d^, Hoang et al. (2019) ^e^, Jovtchev et al. (2006) ^f^, Arata et al. (2015) ^g^, Hara et al. (2013) ^h^, Ladouceur et al. (2015) ^i^, Conklin (1912) ^j^, Maciak et al. (2011) ^k^, Gibeaux et al. (2018) ^l^, Jevtic et al. (2015) ^m^, Jaasma et al. (2006) ^n^, and Tsichlaki and FitzHarris (2016) ^o^. See Fig 2 for the same dataset presented with nucleus:cell ratio on the y axis.


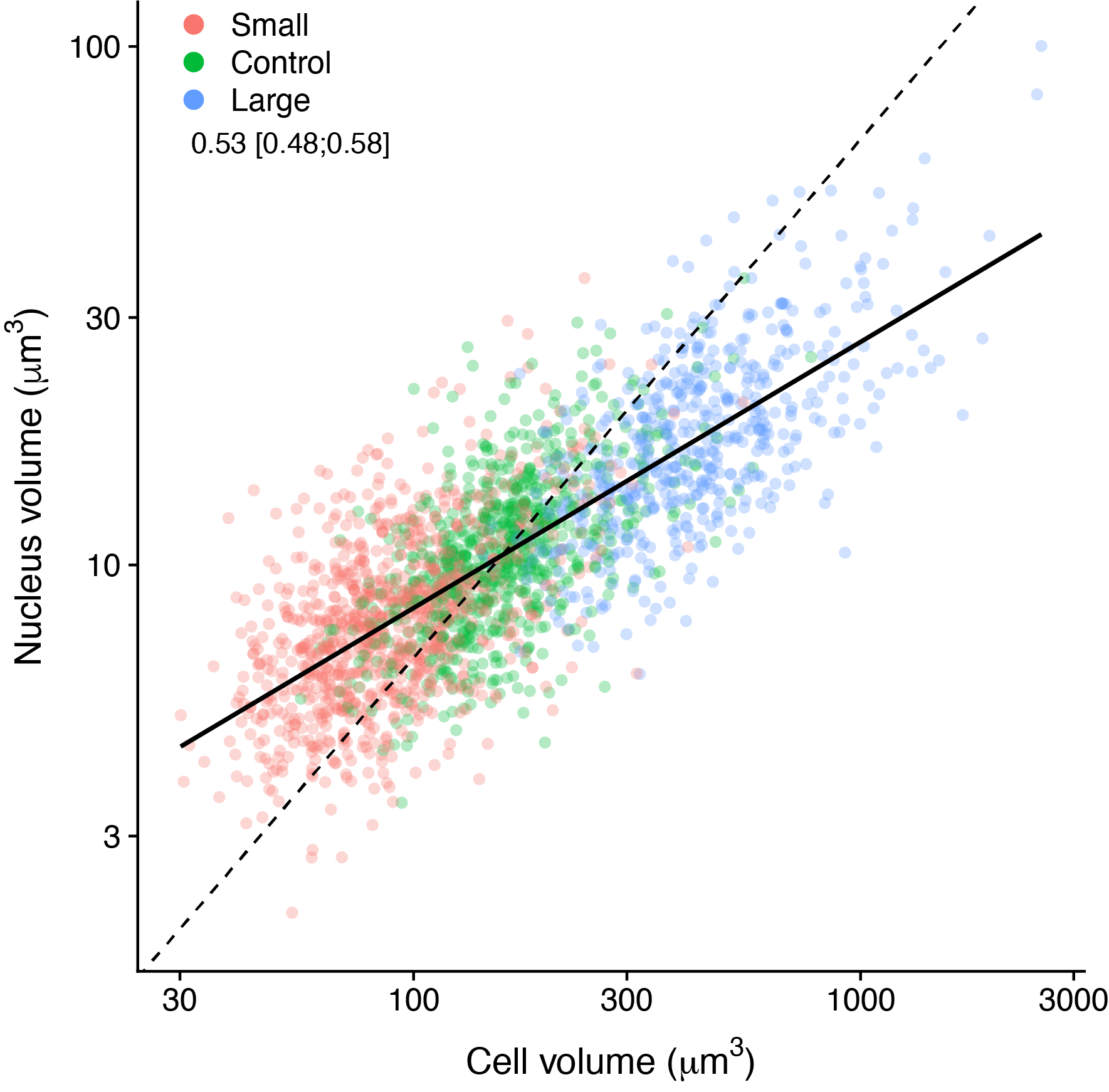


**Figure S9:** Nucleus volume and cell volume among cells of *Dunaliella tertiolecta* that were artificial selected for size (both axes are log_10_-transfromed). Each dot represents a cell and the colour indicates the size-selection treatment. Continuous line shows the fit of a linear mixed-effect model, whose slope coefficient is reported in the legend [$\pm$95% C.I.]. Dashed line displays the null hypothesis of a size-invariant N:C ratio (i.e. slope = 1 and intercept estimated from the data). See Fig. 3 for the same data presented with N:C ratio on the y axis.
